# A theory based evaluation of an intervention to promote positive health behaviors and reduce social isolation in people experiencing homelessness

**DOI:** 10.1080/10530789.2019.1623365

**Published:** 2019-05-30

**Authors:** Stephen Malden, Ruth Jepson, Yvonne Laird, John McAteer

**Affiliations:** Scottish Collaboration for Public Health Research and Policy, School of Health in Social Science, University of Edinburgh, Edinburgh, UK

**Keywords:** Homelessness, physical activity, social isolation, evaluation, program theory

## Abstract

Homelessness adversely affects an individual's ability to access healthcare, opportunities for social interaction and recreational activities such as physical activity. This study aimed to evaluate the impact of a community-based physical activity and peer support intervention on the health and wellbeing of homeless participants. This study employed semi-structured interviews to investigate the perceived impact of the Street Fit Scotland intervention on the health and wellbeing of 10 homeless adults. Interviews were audio recorded and transcribed verbatim. A thematic analysis was conducted. Participants reported that their health and wellbeing had improved since attending the intervention. This was attributed to improvements in self-esteem, social interaction and mental wellbeing. Participants generally felt that their physical activity had increased since attending Street Fit Scotland, and a number of individuals reported that they were making healthier choices with regards to health behaviors. A theory of change logic model was developed that demonstrated how each component of the intervention influences the observed and intended outcomes. Attendance of Street Fit Scotland had positive effects on participant's health and wellbeing, particularly concerning self-esteem, health behaviors, social interaction, and physical activity. More efforts should be made to evaluate small-scale interventions that are reaching vulnerable population groups.

## Introduction

Homelessness is a global problem, and while obtaining an accurate estimate is difficult, a reported 216 million people are homeless worldwide (Tipple & Speak, 2009). While homelessness is a particular problem in the developing world, it also remains a major issue in developed regions, with current prevalence estimates for the USA and Europe being 630,000 and 400,000 respectively (Bernstein, Meurer, Plumb, & Jackson, 2015; Cortes, Henry, de la Cruz, & Brown, 2012; Fazel, Geddes, & Kushel, 2014). In 2015, a total of 28,615 people were identified as homeless in Scotland (Fitzpatrick, Pawson, Bramely, Wilcox, & Watts, 2015), while the total number of reported cases of homelessness in England was 271,000 in 2016 – a rise of 32% since 2010 (Fitzpatrick, Pawson, Bramely, Wilcox, & Watts, 2017). A rising trend in homelessness in the UK has continued through the last decade due to austerity measures (Loopstra, Reeves, Barr, Taylor-Robinson, & Stuckler, 2014), and is forecast to accelerate following budget reforms (Gibb, Sprigings, Wright, & McNulty, 2014). Interventions to reduce barriers and alleviate the financial burden of treating ill health among the homeless are therefore urgently needed (Bernstein et al., 2015; Hwang & Burns, 2014).

People experiencing homelessness are confronted with significant barriers regarding access to basic healthcare (Bernstein et al., 2015; Randers et al., 2012). Such barriers add to a healthcare burden for which the prevalence of communicable and non-communicable diseases is already disproportionate to levels observed in the general population, especially with regards to mental illness (Irwin, LaGory, Ritchey, & Fitzpatrick, 2008; John & Law, 2011).

Interventions to address the communicable diseases commonly associated with homelessness such as tuberculosis and hepatitis C are well established (Tankimovich, 2013). However, those which address the wider issues homeless people face are less apparent. Two such issues are lack of opportunities to be physically active (Wilson, 2005) and social isolation (Matthews et al., 2016; Sanders & Brown, 2015; Steptoe, Shankar, Demakakos, & Wardle, 2013). The potential for physical activity interventions to benefit homeless populations is supported by an abundance of research which has been conducted on the general population. Not only has physical activity been shown to reduce the risk of mortality, heart disease, obesity, type 2 diabetes and osteoporosis (Lee et al., 2012), it is also associated with improved mental health (Hamer, Stamatakis, & Steptoe, 2009; Richardson et al., 2014). Social isolation and stigmatization are a particular problem for homeless people, and can often lead to depression and self-harm if not addressed (Haw & Hawton, 2008; Kidd, 2007). Such issues are highlighted in a retrospective cohort study which demonstrated that homeless populations in Scotland were over three times more likely to die as a result of self-harm than the general population (Morrison, 2009). Given the high prevalence of depression and social isolation among homeless populations (Wright & Tompkins, 2006), interventions that address these issues are needed.

Little is known about the impact of physical activity and peer support interventions on people experiencing homelessness in comparison to other population groups. A mixed-methods review of health interventions for homeless individuals identified no robust randomized trials which had physical activity as a core component (Coles, Themessl-Huber, & Freeman, 2012), and only one qualitative study in which peer-led education was utilized. A recent process evaluation by Sofija, Plugge, Wiseman, and Harris (2018) found that formerly homeless individuals experienced improvements to their overall wellbeing and social inclusion through participation in a group exercise intervention (Sofija et al., 2018). Similarly, Welty Peachey, Lyras, Borland, and & Cohen (2013) evaluation of the street soccer USA cup concluded that the intervention was positively perceived by participants as it created a sense of community and an inclusive environment for social interaction to occur (Welty Peachey et al., 2013). While the aforementioned studies demonstrated the potential for physical activity interventions to improve general wellbeing and social support in homeless individuals, a feasibility study by Kendzor et al. (2017) found that objectively measured moderate-vigorous physical activity (MVPA) significantly increased in homeless individuals participating in a 4 week intervention when compared to controls (Kendzor et al., 2017). However, this area of research is still in its infancy. Therefore, more in-depth understanding of the potential impact of physical activity and peer support interventions on this population is needed, as the potential for such initiatives to improve health and wellbeing has been demonstrated with other population groups (Ekeland, Heian, Hagen, Abbott, & Nordheim, 2004; Greaves et al., 2011; Pfeiffer, Heisler, Piette, Rogers, & Valenstein, 2011).

Robust evaluations are critical to understand if these interventions are effective and how they work, for example by identifying an intervention's theories of change, allowing for further development of the program theory. Theories of change are essentially the mechanisms within an intervention which lead to its intended outcomes (Pawson & Tilley, 1997). This information is often presented visually as a logic model to illustrate how each mechanism is related to – or impacts upon – each outcome.

The aim of this study was to explore the impact of a community-based physical activity and peer support intervention (Street Fit Scotland) on the health and wellbeing of people experiencing homelessness, and to develop a program theory which illustrates how each specific component of the program likely contributes to its functioning.

## Materials and methods

### Design

A qualitative study was conducted for this evaluation.

### Setting

Street Fit Scotland (SFS) is a not-for profit organization which aims to improve the health and wellbeing of people experiencing homelessness in Edinburgh (UK) through the provision of group exercise classes and peer support meetings. The program started in 2015 and runs indefinitely on a weekly basis. The intervention was developed by a homeless-services worker due to their practice, observations, and knowledge of this specific population group. Participants attended an instructor-led group fitness class at a local leisure center once a week (approximately one hour long). The class involved a mixture of aerobic circuits and strength-based resistance training exercises to music. Fitness classes were then followed by a peer support workshop where participants had the opportunity to discuss their situation with a support worker, or socialize with other members of the group and have lunch provided by the SFS staff. The meetings also offered the opportunity to take part in workshops with a Community Psychiatric Nurse or listen to seminars from guest speakers, which aimed to support participants to make informed decisions about their health and wellbeing. While the workshops occasionally had designated themes, they were generally unstructured and allowed the participants to choose the topic of the workshop and direction of discussion each week. At the time of this evaluation, the typical attendance of SFS was 12–15 participants per week. The majority of these participants were regular attendees of the program (had attended >10 sessions), however, others were sporadic in their attendance. The intervention took place in a local authority leisure center (fitness classes) and a community center (peer support workshops) in Edinburgh, UK.

### Sampling and recruitment

A purposive sampling strategy was used to recruit adults who were experiencing homelessness and who were attending both the fitness classes and peer support components of the intervention in March 2016. Participants were sampled based on them being homeless (defined as living in hostel accommodation or on the streets), over the age of 16 years, and had attended at least ten SFS sessions (*n* = 12). Prior to recruitment, the first author attended a number of fitness classes and workshops to build a level of trust and rapport with potential participants. This researcher also conducted the interviews with participants. A member of staff approached each participant at a convenient time during the sessions and asked if they would like to participate in the study. Ten of the twelve regular attendees took part in the individual interviews (five men and five women) with ages ranging from 21 to 36 years of age. All but one of the participants were unemployed.

### Data collection

The interviewer (SM) and secondary data analyst (RJ) are both extensively trained in qualitative research methods and public health program evaluation. Interviews were conducted once with each participant by the first author in a private room at the community center where the peer support workshops were held. All interviews were conducted 12–15 weeks after the researcher first attended the program. A semi-structured topic guide (Appendix 1) was developed by the research team with accompanying prompts and probes. The topic guide consisted of retrospective questions relating to the perceived effects of the intervention on participant's social isolation, physical activity levels, health behaviors, and general physical and mental health and wellbeing. Questions also explored participant's views as to which aspects of the intervention worked well and not so well, in addition to any barriers or facilitators to participation. All interviews were audio recorded before being transcribed verbatim for analysis.

### Data analysis

A thematic analysis (Vaismoradi, Turunen, & Bondas, 2013) was undertaken for this study. All transcripts were analyzed and coded by one researcher (SM), and a second researcher (RJ) independently analyzed 50% of the dataset. Both authors compared their coding, with any discrepancies in the definitions of codes, or the assignment of codes to specific lines of text being discussed and agreed upon to refine the coding framework. Themes were then applied to the dataset using an inductive analytic approach in order to study the ways in which the SFS intervention was influencing participants’ health and wellbeing. Data analysis was facilitated by NVivo 11 qualitative data analysis software. A logic model of the theories of change was then developed using the themes by identifying how each component of the intervention may have influenced the other. Recommended procedures for logic model development were followed, detailed elsewhere (Moore et al., 2015). Briefly, underlying issues facing homeless populations were identified through existing literature and consultation with intervention developers. Planned inputs and their desired purpose were determined, before specific intervention components were determined, and potential mechanisms of impact identified through participant interview responses. Following completion of data analysis, a lay summary report of the findings was presented in a workshop to the participants.

### Ethics and informed consent

This study was granted ethical approval by the Centre for Population Health Sciences Ethics Review Group, University of Edinburgh in August 2015. Informed consent was obtained directly from the participants who completed the consent form with the assistance of a staff member if they had literacy issues. Participants received no compensation or incentives for taking part in the study.

## Results

The participants discussed a number of benefits that SFS was having on their physical and psychological health, as well as barriers and facilitators to participation. A number of the findings map onto the questions presented in the interview topic guide, while others were newly emerging categories from the dataset identified during thematic analysis. The following sections outline these findings in more detail and provide illustrative quotes.

### Health behaviors

Participants described engaging in more positive health behaviors as a result of their participation in SFS. All of the participants stated that their participation in physical activity had increased since joining the program. Participants attributed this to increased confidence, and the provision of a service that in normal circumstances would not be affordable for them (access to a gym). Nine of the participants also reported increases in physical activity outside of the setting of the intervention. Participants stated that they felt more confident to exercise independently, or had realized through participation that physical activity was a behavior they enjoyed, and thus began incorporating it into their daily lives.

we have done all sorts we’ve went on walks, we’ve went cycling up at Ratho [geographic location] and that so we’ve done stuff, and I wouldn't have done it unless I came here [the intervention] because as I say there's nothing else to do so, that got me into physical activity

It also appears that participation in SFS had a positive effect on engagement in other health behaviors. Specifically, nine participants stated that they had reduced harmful health behaviors such as alcohol consumption and drug use, or had made healthy changes to their diet. Reasons for this were increased feelings of self-worth, the formation of supportive social groups, a desire to be in optimal physical condition when going to the gym, and a general increase in health-consciousness among participants.

We are going home for dinner, we even sometimes have dinner together, like we eat together. We are making a lot of healthier choices together as well. Like I can't speak for everybody I’m not saying everybody's stopped drinking … but everyone is supporting each other

I’ve noticed a massive improvement in my fitness, and it's definitely keeping me motivated to live a healthy lifestyle, because you don't put in all that hard work and then want to ruin it, you know what I mean? You don't set yourself up on a day with alcohol or you’re not gonna want to work out, and if you do work out then the alcohol is compromising your experience and your gains and what you actually take away from it

### Self-esteem

All participants indicated that attending SFS had a positive impact on their self-esteem. Specifically, participants stated that having the support of competent, supportive staff contributed to this, as did participation in challenging fitness classes and peer support workshops.

being in the hostel and what-not it did sort of, drop down to zero [confidence], so being here, [staff member] gives you that confidence, she knows that you can do it, she pushes you to do it because she knows you can. So it does it has given me a massive boost

The majority of the participants suffered from extremely low confidence before joining the intervention, which was further complicated by other issues such as social anxiety, depression and substance misuse. However, participants felt that the intervention helped by giving them a sense of routine, and others felt motivated by the physical and social benefits of attending.

it's had a positive effect on me like, just with my confidence … because I suffer from depression and stuff I can be like in the house quite a bit, but with street fit I know I have to get up in the morning on a Tuesday and I know I have to get to the gym

it's made me more motivated and obviously after doing it for a wee while you start seeing small changes, but then that sort of makes you more motivated to carry on and keep going and carry that on and see the bigger changes

### Social support and interaction

A major reason for participants’ continued attendance of the program was the opportunity it presented to interact with other people in a similar situation to themselves. The majority of the participants experienced some degree of social or physical isolation before participating in the intervention, and felt less isolated as a result of attending the program. Participant's definitions of isolation varied, but cited long periods of being alone in the hostel or a lack of access to social interaction when discussing these topics. All the participants stated that one of the main reasons they continued to attend the sessions was to get the opportunity to see friends and people with a shared experience.

you are working out which is good for your body and the mind, you’re meeting new people, going different places, which I wouldn't do if I wasn't at street fit Scotland

we even sometimes have dinner together, like we eat together, which we didn't really do before [attending Street Fit Scotland].

The support appears to be very important during the fitness sessions, where the exercises can become challenging. Participants stated that others in the group would encourage them to continue when they were struggling to keep up, which strengthened bonds in the group and increased confidence levels as previously discussed.

I was sitting at the side I couldn't breathe, I was broken I felt sick, and [participant] came over and said look, if you don't get up now, you’re not gonna get up. And that gave me the momentum to get up and push through

Overall, participants had low opinions of themselves prior to taking part in the intervention, and perceived that their increased self-esteem was partly due to the increased social support they received on the program

Honestly all I think I was back then was just a fat junkie, and that's all I thought of myself as, but then [staff member and participant] made me start thinking positive about myself and street fit has helped keep that going

### Perceived effects on physical health

Eight of the participants felt that the fitness classes, coupled with changes in health behaviors had led to noticeable benefits to their physical health. Participants stated that they had noticed visible changes to their body composition, felt fitter/physically stronger, and that others had commented positively on how they look since attending the program, which has in-turn increased their confidence

I took my measurements when I started street fit, and I took my measurements now, and I’m a lot more buff and you know I feel stronger and fitter.

Well, it's [Street Fit Scotland] definitely had a positive effect on my fitness levels and obviously it's brought my confidence out

### Perceived effects on mental health

Four of the participants stated that they have a diagnosed mental health disorder which they received treatment for in the past. Others reported occasionally suffering from mental health problems such as stress, mood-swings, anxiety, and depression. Attending the program was generally perceived to improve participant's mental health, with the improvements in self-confidence and increased social interaction seen as the main reasons for this

it's started to make me feel a lot more confident, like I said before it's really helping with my social anxiety as well, getting out, meeting new people and just doing normal stuff. I mean I’ve sat with my head in between my legs for about sixteen years, and to be awake and alive and getting the opportunity to come to street fit, I feel privileged

I’d stay in the house all the time, didn't have the confidence to go outside, I felt a lot of like anxiety and this, the gym and stuff helps me with my anxiety really well

Another reason commonly described as leading to improvements in mental health within the group was the increased physical activity due to the fitness classes, as these were perceived as a welcome distraction from issues such as stress and anxiety

oh definitely, it does [help mental health], it takes your mind off things, it does sort of help you get relief, release a little bit of like stress and anxiety and what-not when you are there because you can't think about it

I had a problem with sustaining my moods, like so I was pretty up and down and that quite a lot. But now I’d say it has leveled out because I’m exercising and stuff

### Program content

Participants were generally positive about the program content as a whole, with the exercise classes being perceived as challenging but rewarding. However, the majority of the participants indicated that they felt that they would benefit from more sessions during the week, especially after they began to notice positive changes to their health and wellbeing.

more sessions would be a lot better because sometimes you find yourself, because it is only that one day a week you think oh, maybe its ok if I don't go, and sometimes you can just lose that motivation quite quickly, then you are going aw I can't be bothered going today

All the participants agreed that the workshops were an important aspect of the program and they had gained confidence and knowledge from attending them. However, the majority of the participants stated that various aspects of the workshops could be improved upon. Specifically, it was generally felt that the workshops sometimes lacked a clear focus, and this made it difficult for participants to get something out of the sessions.

I think the workshops should be more focused, maybe even letting people know the week before what's gonna be discussed the next week, and to stay on track and not go off track and take detours and start talking about people who are not here and just keep it focused on one subject instead of jumping between all different subjects

When asked what kind of focus the participants would like to see from the workshops, most participants wanted more sessions directly related to dealing with the various challenges homelessness presents, for example retaining a place to live or managing medication.

I just don't really feel they [the workshops] are applicable to the situation that I’m in myself, I don't think that there's enough talk about what to do after the [Hostel], how you are going to cope when you get your own tenancy, how you are feeling if you’re on a prescription, things like that

Several of the participants also highlighted logistical aspects of the workshops which could potentially be improved upon. It was commonly accepted that after the gym class in the morning, a two-and-a-half hour long workshop was difficult to get through in terms of maintaining focus and concentration, meaning participants were sometimes leaving the sessions feeling drained.

I think when you get to this time of the day after you’ve been-because it is quite an intense class, so after that you do sort of just want to go home and relax, and just lie on your couch

they are quite long like [the workshops], I mean if you have lunch and it's after a fitness class-so you’re a bit knackered, sometimes it can go on a bit if you’ve had a hard session

A number of participants suggested that they would benefit more from the workshops if they took place at the beginning of the session before the exercise class, with the lunch coming at the very end before going home. Participants felt that they would be more clear-minded and able to concentrate if this was the case.

I think sometimes maybe doing the workshops first might be a bit better, and then the gym after

### Staff

Participants only had positive remarks to make about the program staff and volunteers. The majority of participants stated that the encouragement and support they received from the staff and volunteers was one of the main reasons they attended the program. Specifically, the enthusiasm and support shown from the staff was perceived to have a positive impact on the self-confidence of the group.

I think she's amazing [staff member] is like one of a kind she does 150%, she goes above and beyond her job title even at [the hostel]like she goes above and beyond what she has to do. She really enjoys her work and you can see it and I suppose it shines through, like it rubs off on us like she enjoys it, she enjoys taking part with us and she has the faith and the confidence that we can do it, so it then gives us the confidence to do it

The safe environment that is created by the staff members appears to encourage the participants to engage in the program and seek help when they need it.

She has got that empathy with us, she knows what we have been through, like you need people on that same sort of level that aren't going to judge you, because a lot of people in these sort of workplaces like a lot of people stop themselves from getting help because of the people that are giving it

The gym instructors were also perceived to be professional, likeable and motivating, which participants stated was important for keeping them focused during the difficult fitness sessions.

their sense of humour and the fact that they’re setting us nearly impossible tasks, but they’re recognising that and the feedback you get after it when they say like “well done” makes you feel proud that you’ve done it, and the more and more times you get told each week like “well done you’re doing well” you start to believe it and it's just going to make your whole life change

I think the instructors are really bang-on they’re good guys, they’re sociable guys, and they’re approachable. They don't mess about they get in they get it done, it's really a high standard you know the guys are accredited HND’d everything, and you never feel embarrassed to put your hand up and ask them for help or anything the guys put you at ease in that sense, I can't fault any of the staff

Four of the participants stated that having a community psychiatric nurse (CPN) present at the workshops was a valuable addition to the team, as this was seen as a means of increasing access to mental health services, which some participants had experienced difficulties accessing in the past

I’m also getting the chance to work with people, who work with people like myself so like [CPN], he's a CPN and that. So that's also comforting to know as well that there's a support network there. And I know that if I had issues I could speak to [staff member] who's got experience, and [CPN] who's got a lot of experience

**Figure 1.**
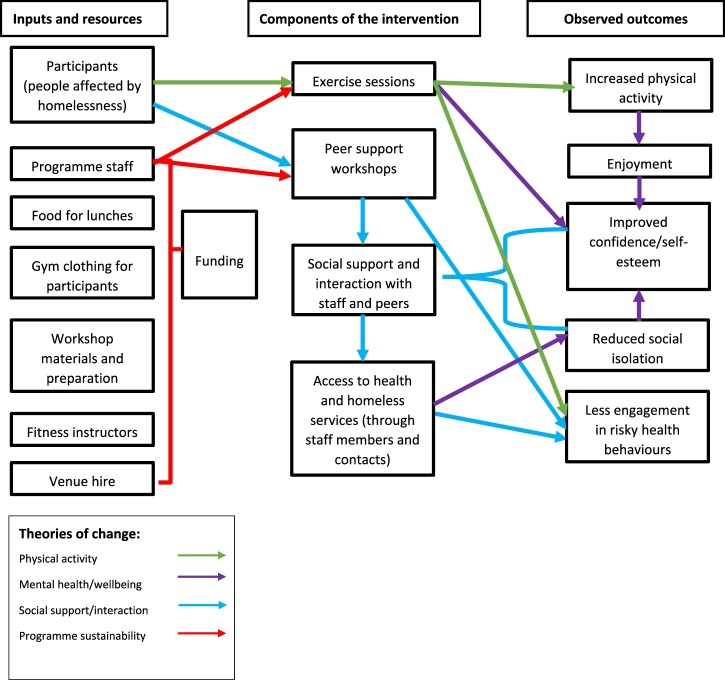
Theories of change logic model for Street Fit Scotland.

Figure 1 demonstrates how each component of the program contributes to its functioning, based on the theories of change identified through the interviews.

## Discussion

Research on homeless populations is lacking throughout the literature, and this appears to be one of the first evaluations of a physical activity and peer support intervention for this particular population group. This evaluation has demonstrated that participation in SFS had a positive influence on people affected by homelessness through the removal of barriers to access physical activity and engage in peer support. Specifically, the intervention appeared to go beyond improvements in physical activity levels as improvements in self-esteem and general mental wellbeing appear evident. This evaluation has also highlighted potential areas for improvement within the content and delivery of specific components of SFS.

The reported improvements in mental wellbeing are likely linked to the other improvements reported by participants for self-esteem and social support and interaction. Fitzpatrick (2017) reported that perceived social support had a protective effect against depression in a cohort of homeless adults (Fitzpatrick, 2017). Additionally, a twin study has demonstrated that socially isolated and lonely young adults were more likely to be depressed than their peers who had regular social support and interaction (Matthews et al., 2016). Considering these observations, it appears that participants in SFS are benefitting from the opportunity to interact with peers and access a socially supportive environment, which in turn may mediate any effects of depression and social anxiety which participants reported to suffer from. This potential relationship is illustrated in the logic model in Figure 1 above. Welty Peachey et al. (2013) also found in their evaluation of the Street Soccer USA cup that the intervention fostered a sense of community and enhanced social networks. Specifically, participants stated that participation allowed them to build stronger relationships with their teammates, and create friendships with other homeless people from different cities who shared similar life experiences and struggles (Welty Peachey et al., 2013).

A particularly interesting finding was that some participants felt that engagement with the intervention was enabling them to reduce certain potentially harmful health behaviors such as binge drinking and drug use, in addition to consciously eating healthier options with the intention of maximizing benefits of engagement in regular physical activity. While the results of this study indicate that this is due to a desire to maximize benefit from physical activity, previous research indicates that a possible link between self-esteem and health behaviors could also have influenced such changes in behavior (Marmot, 2003; McGee & Williams, 2000; Trzesniewski et al., 2006; Veselska et al., 2009). Indeed, participants in the present study reported improvements in self-esteem; therefore, it could be argued that it is the improved self-esteem which is leading to these healthier choices, as opposed to the desire to maximize physical benefits of the exercise classes. Alternatively, it may be a complex interaction between improvements in both physical health/fitness and self-esteem which has been demonstrated in previous research in adolescents (Biddle & Asare, 2011; Ekeland et al., 2004; Fox, 2000; Herman, Hopman, & Sabiston, 2015), but which merits further investigation amongst homeless populations.

Considering the wider benefits reported by participants who have been participating in SFS, it could be argued that the success of the program is mainly attributed to the improved mental wellbeing, self-confidence, and social support as opposed to any reported improvements in physical activity. While improving physical activity is one of the objectives of the program, the overall aim was to target the general health and wellbeing of the participants, not solely to improve physical activity alone. It could be argued that the physical activity component of the intervention is merely a tool to draw vulnerable people to participate, in the hope that they will engage with the activities, and in turn begin to experience the potential wider benefits reported in this study. This raises the question of how the intervention leads to these positive changes to mental health and wellbeing. For example, it could be that providing access to a safe environment and peer support led to these positive effects, rather than the physical activity component of the intervention. Given that other interventions targeting socially isolated individuals with similar structures to SFS have also brought about positive effects on mental health and wellbeing through components such as art and crafts (Greaves & Farbus, 2006) there is a need to better understand the mechanisms through which these interventions bring about change, which could be addressed by future research. This highlights the importance of evaluating grassroots interventions, and helping to identify the various interactions between specific components of the intervention and the outcomes in the form of program theory. The logic model developed in this study could provide a starting point for evaluating or adapting existing interventions, and the development of new ones with similar resources, aims and target population groups (Funnell & Rogers, 2011; Moore et al., 2015; Rogers, 2008).

The findings have identified potential considerations if implementation of SFS more widely is being considered. Firstly, the participants highlighted the peer support workshops as an area that could be improved upon. Sessions that lasted too long caused participants to lose focus, while it was felt that sessions that did not address specific issues related to homelessness were not as engaging. Refining the delivery and content of these sessions is important for any future expansions of the program, as these components of the intervention are likely to contribute to the overall aims of SFS. One interesting point to consider during any future expansions of SFS is the value the participants placed on the staff. Such observations are important to consider when replicating or expanding an intervention, as the personal qualities of those who deliver the intervention can greatly impact on its success, as previous research has demonstrated (Jago et al., 2015; Smith et al., 2017). This observation is also supported by the findings of Sofija and colleagues, as participants in this similar intervention delivered in Australia also placed significant emphasis on the importance of building a good rapport with instructors and having staff who are supportive and non-judgemental (Sofija et al., 2018), a finding that is mirrored in this present study.

There are a number of limitations to this study which should be considered in relation to the findings. Firstly, as with any study focusing on health behaviors and behavior change, social desirability bias may have influenced the findings. Participants may have been reluctant to divulge information regarding engagement in destructive health behaviors, or may have overstated engagement with heathy behaviors. Secondly, due to the small scale of the intervention itself, it was not possible to recruit a large sample size. This, along with the relatively young age of the sample and similar time spent homeless limits this studies generalizability to homeless population groups as a whole. Additionally, it was only possible to conduct interviews with the participants at one-time point, thus making it impossible to assess the longitudinal effects of participation in the intervention. This evaluation used qualitative methods, as the inclusion of quantitative approaches would have been adversely impacted by the small sample size. However, as SFS grows and the potential sample size increases, it would be useful to use a mixed-methods approach in any future evaluation efforts. Specifically, obtaining objective measures of physical activity and wellbeing would further validate any qualitative findings through data triangulation (Creswell & Clark, 2007). We only recruited participants who were regular attendees of the program, and who had built an adequate level of rapport with the researcher. This could have introduced bias into the study, as participants may have believed the researcher to be affiliated with the program. However, we were explicit at the start of our interviews with participants that the researcher was not a part of SFS, and that they could be as honest as they wished during the interviews without any repercussions. The participant's somewhat negative perspectives towards the workshops indicate that this may not have been an issue in this study. Finally, while the logic model we developed offers a visual representation of the findings identified in the study, it is important to highlight that it is a crude representation of the program's theories of change, and may not accurately represent the true causal pathways between the intervention's inputs and outcomes, and can be further refined through future evaluation efforts.

Despite the limitations identified, this study still has a number of strengths. While the sample size is indeed small, it is important to consider that this is a largely new area of research, therefore the findings do increase the knowledge base regarding physical activity and peer support interventions for homeless groups. Furthermore, obtaining rich data from vulnerable groups is notoriously difficult (Aldridge, 2014). The researcher who conducted the interviews with the participants in this present study also attended both the exercise sessions and the peer support groups for ten weeks prior to any interviews taking place. This established a rapport between the researcher and participants, which facilitated the collection of rich data. Evidence of data saturation also became apparent during data collection, indicating the sample size was adequate and efforts to maximize data richness were successful. Finally, the development of a logic model and program theory is particularly useful for grassroots interventions such as SFS, as it allows for the visualization of how each component of the intervention influences particular outcomes, and also acts as a starting point for the development of similar interventions which may have limited resources and similar components (Moore et al., 2015).

## Conclusions

Street Fit Scotland appears to have been successful in meeting its aims to improve health and wellbeing of people affected by homelessness. Participants reported improvements in health behaviors, physical activity levels, self-esteem, and social interaction. Areas for improvement were also identified regarding the workshops, and a logic model of the program theory was developed to assist with future planning and expansion of the intervention.

Conducting robust evaluations of grassroots interventions such as SFS is challenging, but no less important than evaluations of larger, more established programs as smaller scale interventions often reach the most isolated individuals, as this evaluation has demonstrated. Homelessness remains an under-researched area, particularly with regards to the effectiveness of interventions which address the wider issues faced by this hard to reach group. Future efforts should be made to evaluate existing community-based interventions and develop a program theory which can be used by other researchers and practitioners as they attempt to design similar interventions.

## Data Availability

Full transcripts of interview data are available from the corresponding researcher upon reasonable request.

## Appendix 1: Street Fit Scotland interview topic guide

**How did you come to be involved in Street Fit Scotland (SFS)?** (Probe to gain insight into the participant’s circumstances surrounding their initial involvement with SFS, who recruited them, what compelled them to give it a try, how did they initially feel about going along to a session).

**Before you took part in SFS, how would you describe your physical activity levels?** (Probe for recollections of behaviours related to physical activity-or lack of. Find out the reasons that they either were, or were not physically active before this time with a particular focus on barriers and access issues associated with homelessness)

**How has attending SFS changed your participation in physical activity?** (Probe for the ways in which the intervention may have alleviated the barriers previously discussed in question 2, or how it may have increased access-or not. What is it about the intervention that has caused any changes, structure, organised sessions, the support of a group, staff –should all be probed for).

**SFS also includes peer support/ workshop sessions after each fitness class. What are your thoughts on these sessions?** (Probe for ways in which the peer support sessions may add something extra to the experience. Ask what particular sessions or workshops they find most informative/beneficial and why. Do they feel they complement the fitness sessions well?)

How would you describe your overall physical and mental health?

What overall effects (positive or negative) do you feel your participation in SFS has had on your health and general wellbeing? (Probe for any noticeable changes they have experienced or observed regarding their fitness, mental health and wellbeing, and general health since taking part in SFS compared to before. What particular components of the intervention do they feel has caused any changes they have noticed i.e. fitness classes, peer support groups, and why, determine whether they have made any recent changes to their health behaviours, and their reasons for doing so)

**What do you feel works well for you regarding SFS?** (Find out particular aspects/components of the intervention which they are pleased with and feel they benefit from. Also focus on logistical aspects such as the venue, staff and duration/frequency of sessions).

**What do you feel could be done to improve SFS in the future?** (Similar to above, probe for any negatives they found while taking part. Find out how these negatives may have hindered their progress/benefit gained during the programme, and ask them how they feel these issues could be resolved for future intervention delivery.)

**How has taking part in SFS made you feel about yourself?** (How has the intervention changed the way they self-identify, i.e. focus on aspects such as self-worth, confidence, has taking part changed the type of people they now associate with/or feel comfortable associating with now)?
